# Long COVID-19 Pulmonary Sequelae and Management Considerations

**DOI:** 10.3390/jpm11090838

**Published:** 2021-08-26

**Authors:** Afroditi K. Boutou, Andreas Asimakos, Eleni Kortianou, Ioannis Vogiatzis, Argyris Tzouvelekis

**Affiliations:** 1 Department of Respiratory Medicine, “G. Papanikolaou” Hospital, 57010 Thessaloniki, Greece; afboutou@yahoo.com; 2 Critical Care Department and Pulmonary Unit, Evangelismos Hospital, Medical School, National and Kapodistrian University of Athens, 10676 Athens, Greece; silverakos@gmail.com; 3 Physiotherapy Department, University of Thessaly, 35100 Lamia, Greece; ekortianou@uth.gr; 4 Department of Sport, Exercise and Rehabilitation, Northumbria University, Newcastle NE1 8ST, UK; ioannis.vogiatzis@northumbria.ac.uk; 5 Department of Respiratory Medicine, University Hospital of Patras, 26504 Patras, Greece

**Keywords:** Post-COVID sequelae, parenchymal abnormalities, functional limitation, rehabilitation

## Abstract

The human coronavirus 2019 disease (COVID-19) and the associated acute respiratory distress syndrome (ARDS) are responsible for the worst global health crisis of the last century. Similarly, to previous coronaviruses leading to past pandemics, including severe acute respiratory syndrome (SARS) and middle east respiratory syndrome (MERS), a growing body of evidence support that a substantial minority of patients surviving the acute phase of the disease present with long-term sequelae lasting for up to 6 months following acute infection. The clinical spectrum of these manifestations is widespread across multiple organs and consists of the long-COVID-19 syndrome. The aim of the current review is to summarize the current state of knowledge on the pulmonary manifestations of the long COVID-19 syndrome including clinical symptoms, parenchymal, and functional abnormalities, as well as highlight epidemiology, risk factors, and follow-up strategies for early identification and timely therapeutic interventions. The literature data on management considerations including the role of corticosteroids and antifibrotic treatment, as well as the therapeutic potential of a structured and personalized pulmonary rehabilitation program are detailed and discussed.

## 1. Introduction

On 12 March 2020, the human coronavirus disease 2019 (COVID-19) outbreak was declared as a pandemic by the World Health Organization (WHO) [1], and almost one and a half years later, COVID-19 remains the major infectious disease concern globally, causing significant morbidity and mortality [2]. Early in the pandemic, adults with non-severe COVID-19 were thought to have a short-term course of acute illness lasting a couple of weeks, after which symptoms resolved. However, a growing body of literature indicates that there is a substantial minority of patients who may present with persisting clinical features lasting for more than 6 months [3]. While a clear definition of long COVID-19 is still evolving, the National Institute for Health and Care Excellence (NICE) has suggested the following terminology: (a) “Acute COVID-19”, for signs and symptoms of COVID-19 lasting up to 4 weeks, (b) “Ongoing symptomatic COVID-19”, for signs and symptoms of COVID-19 lasting 4–12 weeks, and (c) “Post-COVID-19 syndrome”, for sequelae that develop during or after a SARS-CoV-2 infection and persist for more than 12 weeks; Ongoing symptomatic and Post-COVID-19 syndrome may be both included in the term “Long COVID-19” [4].

Long COVID-19 syndrome is multifactorial and encompasses multi-organ sequelae beyond the acute phase of the infection [2]. It ranges from physical and cognitive abnormalities to functional limitation and exercise impairment, hence leading to the deterioration of quality of life [2]. A wide spectrum of pulmonary manifestations including dyspnea on exertion, restrictive pulmonary physiology, and reduction in diffusion capacity as well as fibrotic lung lesions in HRCT have been reported in up to 35% of survivors directly related to the severity of acute illness. This observation is in agreement with data emerging from severe acute respiratory syndrome (SARS), Middle East respiratory syndrome (MERS), or H1N1 influenza survivors [1].

To this end, and considering the rapidly growing number of COVID-19 cases, identification, documentation, investigation, and management of these long-term consequences using a multidisciplinary approach, represent an imperative need [2]. While a growing body of evidence indicates that a carefully designed pulmonary rehabilitation program may be beneficial for patients with long-lasting dyspnea, fatigue, and exercise limitation [4], the optimal treatment approach of other persisting imaging, functional, and clinical manifestations is less well understood.

The scope of this review is to summarize the current state of knowledge on the (a) risk factors, outcome measures, and management options among COVID-19 survivors who develop fibrotic-like features, (b) clinical features and mechanisms of functional and physiologic limitation in patients with persistent post-acute COVID-19 manifestations, and (c) basic principles, structure, and outcomes of a pulmonary rehabilitation program that should be offered to these patients. Documentation of the above data will increase awareness of post-COVID-19 sequelae and fuel clinical studies addressing patient care needs and management options beyond the acute phase of the disease. It is more than evident that a new clinical entity will arise within the foreseeable future and the development of relative infrastructure and human resources to provide multispecialty care for this subgroup of patients is of paramount importance.

## 2. Risk Factors and Outcome Measures for Post-COVID-19 Interstitial Lung Abnormalities

Post-hospital discharge care of patients with a history of SARS-CoV-2 infection has been recognized as a major research priority and guidance for the management of COVID-19 survivors is still evolving [5]. A broad spectrum of pulmonary manifestations, ranging from dyspnea to strenuous ventilator weaning and fibrotic lung disease, has been reported post-COVID-19 [6]. The concept of early disease identification in COVID-19 survivors at risk for pulmonary fibrosis seems rationale given prior experience with idiopathic pulmonary fibrosis [7]. Extending durable benefits of timely anti-fibrotic initiation to those in the earliest stages of post-COVID-19-interstitial lung disease (ILD) will hopefully reduce both morbidity and mortality of post-acute COVID-19 sequelae [8]. Therefore, early detection of interstitial lung abnormalities (ILAs) is of paramount importance. It is also equally important to timely predict the clinical significance of these ILD patterns and differentiate non-fibrotic from fibrotic-like changes. Current studies report that approximately 5% of COVID-19 patients are left with persistent radiological, physiological, and functional deficits [9]. Previous pandemics by similar coronaviruses, including those of SARS and MERS, reported comparable percentages with ILAs being present in 4.6% at one year and in 3.2% of patients following 15 years from infection [10,11,12]; yet, the proportion of patients with COVID-19 developing residual fibrotic-like ILAs remains to be determined in the context of large prospective studies.

Even minimally symptomatic patients may have objective abnormalities in high-resolution computed tomography (HRCT) following recovery from acute infection. Older age, male sex, acute disease duration and severity, as indicated by the persistence of dyspnea and tachycardia, radiographic disease extent, mechanical ventilation, and prolonged hospitalization have been identified as negative prognostic factors for fibrotic ILD development [2,13,14,15,16,17]. In particular, serial radiographic evaluation of 114 hospitalized patients with COVID-19 showed residual CT abnormalities in 62% of patients 6 months post-COVID-19 [13]. Initial features predictive of residual fibrotic-like abnormalities include older age (greater than 50 yrs), and markers of disease severity (dyspnea, tachycardia, the extent of radiographic abnormalities, prolonged hospital stay, and need for mechanical ventilation) [13]. Patients with persistent dyspnea presented with greater restriction on spirometry, lower diffusion capacity for carbon monoxide (DLCO), and increased exertional desaturation [14]. A previous study reported that 61% of patients with a disease duration of greater than 3 weeks developed persistent post-COVID-19 lung parenchymal abnormalities [18]. The latter statement is significantly premature and potentially flawed as it is currently unknown whether these fibrotic-like features represent a truly irreversible disease in a post-ARDS setting or simply reflect areas of immature fibrosis highly likely to regress. Lack of knowledge on pre-existing ILD poses significant limitations to the above observations [19]. The contribution of COVID-19-induced autoimmunity in ILD development remains to be determined.

To address issues raised above, the British Thoracic Society (BTS) published an algorithm suggesting clinical assessment and chest X-ray in all patients at 12 weeks, combined with pulmonary function tests (PFTs), 6-min walking test (6MWT), sputum sampling, and echocardiogram based on clinical judgment [20]. Based on this 12-week assessment, patients could be further recommended to be evaluated with HRCT, computed tomography pulmonary angiogram or echocardiogram, or discharged from follow-up. Caution should be applied when CXR is performed to monitor ILAs, as ILD practitioners are aware of the deceptive nature of CXR in evaluating ILDs. Finally, an assessment of the quality of life should be also performed considering the negative impact of ILD on parameters of quality of life. A recent study demonstrated that 44% of COVID-19 patients, at 60 days of follow-up, experienced a significant decline in quality of life, as assessed by the EuroQol visual analog scale [21].

Collectively, given that radiographic abnormalities may be present even in asymptomatic individuals, it seems rationale that all patients should have a baseline radiographic (HRCT) and functional screening 2–3 months following SARS-CoV-2 infection. A second functional and radiographic follow up at 6 months could be performed in a substantial minority of individuals with features predictive of residual ILAs including age (>50 yrs), fibrotic-like CT features at first follow-up (parenchymal bands, irregular interfaces, traction bronchiectasis, and/or honeycombing), and markers of disease severity including persistent dyspnea on exertion, tachycardia, prolonged hospital stay (>2 weeks) and history of non-invasive and/or invasive mechanical ventilation. The assessment of patient-reported outcomes using Borg and modified Medical Research Council (mMRC) dyspnea scales, as well as health-related questionnaires, including the St George’s Respiratory Questionnaire and/or King’s Brief ILD Questionnaire, should be also pursued. Immunologic profile investigation should be restrained to those with persistent symptomatology suggestive of an autoimmune disease.

## 3. Treatment Options of Post-COVID-19 ILAs

A substantial minority (almost 40%) of COVID-19 patients experience persistent physiological, functional, and/or radiological impairment extending beyond 2 months of primary infection. Radiographic patterns can be sub-divided into non-fibrotic and fibrotic-like. Non-fibrotic features are predominantly features of organizing pneumonia (ground glass and consolidative opacities), hence the rationale for using steroids in this subgroup of patients. Treatment with oral corticosteroids for 3 weeks led to significant improvements in clinical (dyspnea), functional (increase in forced vital capacity (FVC)%pred and DLCO%pred), physiological (increase in 6-min walking distance (6MWD)), and radiological (ground-glass opacities and perilobular consolidation) in a limited cohort (n = 30) of patients [9]. The optimal dose and duration of glucocorticoids for the management of post-COVID-19 ILAs remain to be determined. An RCT with two arms of different doses of corticosteroids (40 mg of with gradual tapering or a stable dose of 10 mgr prednisolone per day) for 6 weeks is currently ongoing and results are greatly anticipated (NCT04657484). Corticosteroids have shifted the therapeutic dial during the acute phase of COVID-19 [22]; yet, their efficacy in the chronic persistent post-COVID19 syndrome warrants further investigation. It is questionable whether corticosteroids could reverse fibrotic-like lung parenchymal changes and prevent their progression to irreversible fibrosis. On the other hand, caution should be applied to the use of glucocorticoids as they may be a double-edged sword in this clinical situation, particularly in light of the detrimental effects of high doses of oral corticosteroids in patients with IPF [23]. Steroids reduce inflammation associated with organizing pneumonia with a resultant resolution of symptoms, hypoxemia, and potentially early parenchymal abnormalities. However, they are associated with adverse effects such as hyperglycemia, delayed viral clearance, and increased susceptibility to infections, and most recently thromboembolic disease [24].

With regards to the use of anti-fibrotic agents (pirfenidone and nintedanib) for the treatment of post-COVID19 ILAs, data is still scarce and exploratory. Novel anti-fibrotic agents display pleiotropic therapeutic effects including anti-inflammatory and cytoprotective and thus justify the rationale for the treatment of COVID-19-induced lung injury [25]. The safety and efficacy of pirfenidone are currently being investigated during the acute phase of COVID-19 in a population of COVID-19-induced severe ARDS both through the nasogastric tube (NCT04653831) or via inhalation (NCT04282902). It remains to be addressed whether prolonged use of anti-fibrotic and anti-inflammatory compounds may reduce the progression of parenchymal lung abnormalities to irreversible fibrosis. On the other hand, the beneficial effects of antifibrotics in non-IPF progressive fibrosing ILDs support the investigation of their role in COVID-19-induced pulmonary fibrosis [26,27,28,29]. Importantly, patient recruitment in clinical trials investigating the role of nintedanib and pirfenidone in the treatment of SARS-CoV-2 induced pulmonary fibrosis is ongoing (NCT04541680, NCT04607928) and the results are eagerly awaited. These trials will shed further light on the role of antifibrotics in COVID-19-induced pulmonary fibrosis. The rationale for using antifibrotic therapy should be personalized and applied on the basis of persistent clinical, functional, and radiological impairment, as stated above. Many of the current and emerging antifibrotic drugs could have therapeutic potential for treating severe COVID-19 and preventing the long-term fibrotic consequences that might follow this pandemic.

Taken together, there is no established regimen for the treatment of post-COVID-19 ILAs. Various therapeutic strategies including oral corticosteroids with or without anti-fibrotic compounds are under evaluation in the context of large randomized controlled clinical trials (RCTs). Optimal doses and the duration of treatment are unknown and data is still scarce and investigational.

## 4. Respiratory Function Abnormalities Post-COVID-19 Pneumonia

Previously published data suggested that a proportion of patients who recovered from SARS-CoV-2 infection may present with abnormal respiratory function and impaired exercise capacity during short-term follow-up [1]. However, most of these data come from studies among patients who were hospitalized due to pneumonia and/or respiratory failure, so they cannot be generalized in the vast majority of patients with milder disease, treated in the community. In the largest study in the field, reduced DLCO was found to be the most common respiratory function abnormality at six months follow-up after discharge; this was present in 22–56% of patients, depending on the severity of respiratory failure they established during hospitalization [30]. Similarly, in the Swiss observational COVID-19 lung study, the only parameter that predicted the DLCO reduction in the multivariate model was the presence of severe/critical disease [31]. Most patients had normal spirometry at follow-up; nevertheless, when the pulmonary function was abnormal, a restrictive pattern, as indicated mainly by the evaluation of total lung capacity (TLC), or by both TLC and FVC, was the second most common finding [30,31].

These results are in agreement with earlier studies in the field, where DLCO impairment, alone or in combination with a restrictive pattern, was the most frequent abnormality encountered among discharged patients recovering from SARS-CoV-2 infection. However, in these studies, patients were either assessed at the time of discharge or within a month, so the duration of follow-up was very short [32,33]. Notably, even in the presence of abnormally low DLCO and lung volumes, the reductions were mild or moderate in the majority of patients. Resting arterial blood gases were largely normal during follow-up [31,33,34], but mild exercise-induced hypoxemia could be present, especially among patients with severe disease [31]. Finally, although data regarding respiratory muscle function are extremely limited, maximum static inspiratory and expiratory pressures were below normal in a significant proportion of hospital discharged patients during early follow-up [30], but more studies are needed towards this direction.

In conclusion, most patients who recover from SARS-CoV-2 infection present with normal respiratory function during follow-up. In case abnormalities are noted, the most common impairment is decreased DLCO, followed by a restrictive pattern; thus, utilizing simple spirometry for patient follow-up is not adequate, especially for those with previous severe/critical disease. A previous meta-analysis among survivors of SARS and MERS indicated that reduced DLCO may persist up to 12 months after recovery [35]. Thus, longitudinal studies with repeated evaluation of respiratory function variables and a long follow-up period are urgently needed, to determine the pattern of respiratory function changes in time and whether the noted abnormalities may be temporary or persistent.

## 5. Persistent Functional Limitation and Mechanisms of Establishment

Several studies from the United States, Europe, and China have reported persistent symptomatology and functional impairment among patients who recovered from COVID-19 disease [2]. These clinical features may be ongoing, worsening, or even new, after temporary recovery from the acute disease phase [4]. Fatigue and dyspnea have been recorded as two of the most common persistent symptoms [2], while exercise impairment may also be evident in a significant proportion of patients. In one of the first COVID-19 follow-up studies conducted in China, patients with severe disease presented with significantly shorter 6MWD, than patients with mild/moderate disease [32]. Similarly, in another interventional study, elderly COVID-19 patients with comorbidities presented with significantly decreased 6MWD after hospital discharge [36]. Likewise, when 58 COVID-19 patients were assessed 2–3 months after hospital discharge with 6MWT, they covered a significantly shorter 6MWD, compared to the group of 30 matched controls, while almost two-thirds of the patient population presented with dyspnea [37]. Similarly, in the national prospective observational Swiss COVID-19 lung study, patients with severe/critical disease presented with impaired exercise capacity and 120 m shorter 6MWD, compared to the patients with non-severe disease, at four months follow-up [31]. In a larger study in the field, which included 1733 discharged COVID-19 patients, the median 6MWD was less than the lower limit of the normal range in 29% of patients with disease severity of the highest scale (5–6), while the odds ratio for fatigue and muscle weakness was 2.69 (1.46–4.96) for patients with severe/critical disease compared to those with less severe disease [30].

Exercise impairment was also evident in studies assessing cardiopulmonary exercise responses among patients who recovered from COVID-19, although data is currently limited. In a preliminary study of 10 post-COVID-19 patients who were assessed early after hospital discharge, all presented with decreased peak oxygen uptake (peak VO_2_) [38]. Nevertheless, only two out of ten had abnormal ventilatory equivalents for carbon dioxide at the anaerobic threshold, indicating that extra-pulmonary factors were the main cause for impaired exercise capacity [38]. In the study of Baratto C et al., peak oxygen consumption was reduced by 30% among ready-to-discharge COVID-19 patients without major comorbidities, compared to controls, while the two groups presented with a similar respiratory reserve and unaffected pulmonary vascular function [39]. In the largest study in the field, 75 patients with COVID-19 of various severity were recruited, approximately 3–4 months after discharge; of these, 55% established a reduced peak VO_2_. Patients with exercise impairment presented with a significantly lower anaerobic threshold, peak oxygen pulse, slope of oxygen uptake to work rate relationship, and peak work rate, compared to the rest, but they had similar peak ventilation and ventilatory reserve, compared to patients without exercise limitation [40]. No differences were noted in age, disease severity, and respiratory function parameters between the groups with normal and reduced exercise capacity. Interestingly, rest and peak symptoms were comparable. Specifically, more than half of the patients reported dyspnea during daily activity, and the severity of peak dyspnea was similar between those with normal or reduced exercise capacity [40]; thus, additional exercise testing parameters are needed to investigate residual dyspnea among these patients.

Several pathogenetic mechanisms, which are mainly extrapulmonary, have been proposed for the exercise impairment which is evident among patients who recovered from COVID-19. Despite parenchymal lung disruption and endothelialitis, pulmonary vascular function is not impaired after COVID-19 [40]. Similarly, although a restrictive pattern is frequently evident, a respiratory limitation is not established during maximum exercise [39]. At peak exercise, a further increase of oxygen uptake is mainly driven by cardiac output in COVID-19 patients, gradually reaching an oxygen extraction limit [39]. This phenomenon may reflect myopathic changes, either because of medications administered (mainly steroids) or as a direct effect of SARS-CoV-2 on muscle tissue [39,40]. Persistent autonomic derangement, frequently seen after COVID-19 recovery [41], may also impair the distribution of cardiac output to exercising muscles, thus contributing to low peripheral oxygen extraction [39]. Moreover, deconditioning due to prolonged hospital stay and post-hospitalization syndrome [40] may further impair exercise performance, resulting in peripheral limitation before exhaustion of ventilatory reserves, in these patients.

In conclusion, functional limitation and exercise impairment may complicate the recovery course of COVID-19. The presence of comorbidities and severe/critical COVID-19 disease have been reported as potential risk factors, although this finding was not universal when exercise capacity was assessed with CPET. The assessment of functional capacity utilizing an exercise testing should be conducted in the follow-up of COVID-19 patients, especially of those with long hospitalization, severe/critical disease, persisting respiratory function abnormalities, and/or resting symptomatology. Further studies are needed to investigate the characteristics of these patients that are of high risk to establish functional impairment and to determine the duration of this complication.

## 6. Patients with COVID-19 Pneumonia in the Need of Rehabilitation

Patients recovering from COVID-19 infection, regardless of whether they were treated in hospital or not, suffer from a significant proportion of various symptoms, including fatigue and shortness of breath 3 months after the onset of symptoms [42]. In a mixed population of patients hospitalized in Italy due to COVID-19 infection on average for 13.5 days (with 73% having pneumonia, 15% receiving non-invasive mechanical ventilation-NIV, and 5% invasive mechanical ventilation-IMV), only 12.6% were free of COVID-19 infection-related symptoms following re-assessment within an average of 60 days. Thirty-two percent (32%) of patients had 1–2 symptoms and 55% had 3 or more. Forty-four percent (44%) of patients had a worse quality of life (based on the EuroQol visual analog scale). Symptoms included fatigue (53.1%), shortness of breath (43.4%), joint pain (27.3%), and chest pain (21.7%) [43]. Patients treated in a specialized COVID-19 unit in France, with several having been referred to the intensive care unit (ICU), were found to have symptoms and impaired quality of life 110 days after admission. Specifically, 55% of the patients showed fatigue, 42% shortness of breath, 34% memory loss, 28% concentration disorders, and 30% sleep problems. No differences were found between patients admitted to the ward and the ICU, except for increased pain in the latter [44]. A similar telephone survey in England found increased exhaustion, dyspnea, and psychological distress, in patients recovering from COVID-19 illness 4 to 8 weeks after discharge. However, patients admitted to the ICU had an increased burden compared to those admitted to the ward [45]. In patients who were transferred to a rehabilitation center after hospitalization due to COVID-19 infection, despite the relatively small percentage of those who received mechanical ventilation (8.7% non-invasive and 11.7% invasive), there was a significant reduction in physical function and the ability to perform daily activities even when discharged from the center [46]. In a cohort of 1077 patients, of whom almost 1/3 (27%) were admitted to the ICU, only 29% fully recovered, 5 months after discharge. Among the factors associated with poor recovery were two or more comorbidities and a more severe acute illness [47]. In a recent study from China in 1733 patients of whom only 122 needed high oxygen mixtures or mechanical respiratory support, 76% had at least one symptom, 6 months after disease onset. More specifically, 63% had fatigue and muscle weakness, 26% had difficulty sleeping, 23% had anxiety and depression, and symptoms were more intense in patients who needed high oxygen mixtures or intubation [30]. Symptoms extending to 9 months after the onset of the disease have recently been described in 30% of a cohort of patients recovering from COVID-19 infection [48]. Among the suggestions in the aforementioned papers [30,42,45,46], the need to establish rehabilitation programs for patients recovering from COVID-19 disease stands out, especially for those suffering from more symptoms and disabilities.

Based on the aforementioned research and the experience of clinicians battling the COVID-19 pandemic, medical associations worldwide [49,50,51,52] have begun to issue guidance proposing the organization of rehabilitation programs with specific characteristics that will meet the needs of patients who have survived and are recovering from COVID-19 infection. Recently published studies showed that post-COVID-19 patients who are recruited in rehabilitation programs show significant functional improvements [53,54].

Candidates for inclusion in a rehabilitation program are all patients recovering from COVID-19 disease and suffering from a limitation of physical activity, reduction of quality of life, and associated symptoms including shortness of breath, fatigue, weakness of the upper and lower extremities, post-traumatic stress, pain, etc. Various studies show recurring high rates of symptomatic patients who have never been hospitalized, with symptoms that extend as far as 9 months after the initial diagnosis [30,48]. However, patients who were hospitalized, especially those admitted to the ICU, were intubated or received high oxygen mixtures, had more severe symptoms upon recovery [30,45]. Similarly, 14 to 21 days after the positive COVID-19 test only 39% of the symptomatic patients who were hospitalized had recovered their quality of life compared to 64% of those who were not hospitalized [55]. It is known that although two-thirds of the patients admitted to the ICU due to respiratory failure had moderate to good recovery of their physical function within the first two months, the rest who had minimal or no improvement were older with longer hospitalization [56]. Due to the large number of candidates for rehabilitation and the finite possibilities of rehabilitation programs, priority should be given to patients suffering from more symptoms and disabilities. These may include:

1. Patients admitted to the ICU, especially those who were intubated.
2. Patients who needed high oxygen mixtures (high-flow nasal cannula-HFNC and/or non-re-breathing mask) as well as those who were older and had prolonged hospitalization.

Patients in these categories should be screened as a matter of priority for persistent symptoms, poor quality of life, and physical de-conditioning. Various medical associations confirm that these patients are more likely to need and might have the greatest benefit from rehabilitation services [49,50]. If there is availability and adequate resources, patients with milder COVID-19 disease that were treated as outpatients should also be screened. Due to the large number of patients recovering from COVID-19 pneumonia, more funds and recourses should be invested in rehabilitation programs.

Functional impairment can be assessed at 4 weeks of discharge with tools already proposed to assess recovery from COVID-19 disease such as the COPD assessment tool (CAT) [57] and the post-COVID-19 functional status scale (PCFSS) [58]. A CAT score of ≥ 10, or even a PCFSS of ≥ 2, could further distinguish patients who might benefit from a rehabilitation program. New patient assessment tools during and after COVID-19 infection, such as the COVID-19 rehabilitation needs survey (C19-RehabNeS), are constantly being developed to assess functional limitations and patient rehabilitation needs [59].

In conclusion, a significant proportion of patients recovering from COVID-19 infection have symptoms and a worse quality of life 9 months after the onset of the disease. Depending on the resources available, patients recovering from COVID-19 infection should be screened, especially if they (1) have been admitted to the ICU, (2) were intubated, (3) are elderly, (4) had prolonged hospitalization, or (5) have been given high oxygen mixtures. The CAT and the PCFSS can help health professionals identify patients that might benefit from a rehabilitation program. Patients suffering from other diseases and in need of specialized rehabilitation services should be referred to the respective programs.

### Patients Who Should Be Excluded from COVID-19 Rehabilitation Programs

Not all patients that are recovering from COVID-19 pneumonia are eligible for a special COVID-19 rehabilitation program. Patients who suffer from active disease should not be included until they stop being infectious. Patients treated with COVID-19 infection, where the main reason for hospitalization was not the virus infection (e.g., due to coronary heart disease, surgery, etc.), should be excluded. Patients suffering from dementia, chronically paralyzed, with paraplegia, multiple injuries, or other serious orthopedic problems that cause disability or suffer from very serious underlying diseases such as end-stage cancer, and patients with neurological diseases that cause disability, require specialized rehabilitation clinics and special interventions (speech therapy, kinesiotherapy, etc.).

## 7. The Main Features of the Rehabilitation Program

### 7.1. Initiation of the Program

Both the BTS and the joint European Respiratory Society (ERS) and American Thoracic Society (ATS) taskforces recommend that patients perform low-intensity exercise, such as daily activities, for the first 6 to 8 weeks after discharge from the hospital. The working groups consider that high-intensity exercise earlier than 6 to 8 weeks raises multiple safety issues [49,50].

The risk of transmitting the virus and the need to comply with local physical distance regulations create difficulties for the early implementation of outpatient rehabilitation programs. It is unknown whether unsupervised high-intensity exercise programs immediately after hospitalization are safe, especially if there has been no prior formal assessment of exercise capacity [50]. Among the findings of patients recovering from COVID-19 infection are diffusing capacity disorders, which show significant improvement over time [60], as well as parenchymal infiltrates on chest CT scans. Exercise-induced hypoxemia should therefore be expected by health care professionals involved in rehabilitating patients with COVID-19 infection, especially early in a patients’ recovery. Other considerations refer to the possibility of thromboembolic disease or cardiomyopathy [49,50], as discussed elsewhere.

### 7.2. Safety Precautions

Infection prevention and control measures: The necessary protection measures must be taken into consideration to avoid the spread of the virus in the facilities where the patients’ evaluation takes place. These measures are described in detail by the Hellenic National Public Health Organization (HNPHO) [61]. Although exercise is not, according to HNPHO recommendations, included in aerosol-producing activities, patients are not always possible to wear masks during exercise tests, droplets are produced, and the air is potentially polluted. According to recommendations, a naturally ventilated room (open door and windows) requires one hour between patients undergoing non-aerosol producing procedures (half the time when the air is actively circulated 10–12 times per hour via an air pump) to clean the air [62]. Therefore, the evaluation of patients should be done in adequately ventilated areas with each patient individually and a time separation of 1 h. The above predictions must be followed at least in times of a pandemic outbreak.

Exclusion of active infection: Patients upon arrival should be screened for active disease. Fever, new onset of cough, increased sputum, new myalgias, runny nose, and other symptoms compatible with an active respiratory tract infection should be reported and patients referred to appropriate health care facilities for further investigation. Based on HNPHO instructions, the criteria for discontinuation of precautionary measures in a patient with (life-threatening) COVID-19 infection who was discharged from a hospital are: (a) A minimum of three days after the fever subsides (without the use of antipyretics) and respiratory symptoms are improved, and (b) a minimum of 14 to 20 days after the patient stays isolated or two consecutive negative molecular detection tests of the virus in secretions of the respiratory tract with a difference of 24 h between sampling [61]. In other countries, the relevant instructions of local public health organizations should be followed.

Control of complications from the disease: The BTS has published a series of possible complications of the COVID-19 infection that must be taken into account during the initial evaluation of patients who will be admitted to the rehabilitation program [49]:

(a) Patients’ medical records should be checked for thromboembolic disease. Patients should be also checked for symptoms compatible with a new episode such as chest pain, shortness of breath, hemoptysis, new swelling of lower extremities (especially unilateral), arrhythmia, palpitations, dizziness, etc. If COVID-19 infection is complicated by a known thromboembolic disease, at least 4 weeks of anticoagulant therapy should be completed before the initiation of rehabilitation services. Patients taking anticoagulants should be asked if there has been a recent episode of bleeding.
(b) Patients should be evaluated by a cardiologist with an electrocardiogram (ECG) and heart ultrasound and, if necessary, with a rhythm Holter if there are signs of arrhythmia, before joining the rehabilitation program. Patients with myocarditis should not be included in a rehabilitation program for at least 6 months. Patients with coronary heart disease should be included in a rehabilitation program only if the treating cardiologist provides permission.
(c) All patients should be checked for hypoxemia during exertion, as thoroughly discussed elsewhere.
(d) Patients should be checked for new neurological impairments and the risk of falls should also be assessed.
(e) Clinical facilities where supervised rehabilitation programs are performed should be equipped with appropriate safety equipment and staff must be trained in first aid.

### 7.3. Assessment of the Impact of the Disease on Recovery

According to the BTS guidance [49] and the joint ERS/ATS task force statement [50], the recommended assessment of patients recovering from COVID-19 infection is based on a previously published consensus regarding the core outcome measures of patients recovering from ICU hospitalization due to respiratory failure [63], clinical experience, and the special needs of patients recovering from COVID-19 pneumonia. In detail, the proposed assessment tools are the following:

(a) Assessment of quality of life: The European Quality of Life Group-5 Dimensions-5 Levels Questionnaire (EQ-5D-5L) and the Short-Form 36 Questionnaire (SF-36) could be used.
(b) Assessment of emotional function using psychometric questionnaires (such as the Hospital Anxiety Depression Scale and Beck questionnaire) and post-traumatic stress assessment (using, for instance, the Impact of Event Scale-Revised (IES-R) for DSM-IV). Assessment of patients by the department’s psychologist (if available) is also recommended.
(c) Assessment of mental capacity (e.g., with Montreal Cognitive Assessment (MoCA) questionnaire).
(d) Evaluation of the intensity of dyspnea (e.g., with the Modified Medical Research Council Scale, mMRC).
(e) Fatigue severity assessment (e.g., with the Fatigue Severity Scale, FACIT-fatigue Scale, Chalder fatigue scale, etc.).
(f) Assessment of physical activity level (e.g., with the International Physical Activity Questionnaire, IPAQ).
(g) Balance check (e.g., with the Activities-specific Balance Confidence (ABC) scale).
(h) Checking the employment status. Many patients recovering from COVID-19 pneumonia are of working age. Recording what best describes the current employment situation of the patient may include the following: Unemployed-currently in healthcare, unemployed and not looking for a job, unemployed and looking for a job, going to school, volunteer, home/childcare/elderly care, new retirement, receiving new disability payments, awaiting new approval of disability payments, etc.
(i) Evaluation of the functional capacity and capacity for exercise. Tests that can be used include: the Short Physical Performance Battery, the 6MWT, the Incremental Shuttle Walking Tests, the one-minute test–60 s sit to stand test, muscular strength tests of lower and upper limbs (e.g., using a dynamometer), the CPET, etc.
(j) Pulmonary function tests including diffusion capacity.
(k) Body composition check: Measurement of body composition (body fat measurement) and evaluation of the parameters of body mass index, body fat, and muscle mass should be performed. Assessment and consultation of patients by the department’s dietician (if available) is recommended.
(l) Additional assessments: Dysfunctional breathing may require referral to a physiotherapist with specialized skills in this area. Speech and swallowing problems may require referral to a speech therapist. Various orthopedic problems that may arise as a consequence of prolonged prone position (e.g., shoulder dislocation) may require referral to an orthopedist. Peripheral neuropathy may require referral to a neurologist. Symptoms suggestive of post-traumatic stress disorder (PTSD) may require referral to a psychologist. Lack of smell, taste/appetite may require referral to a dietitian. Fatigue can benefit from being referred to an occupational therapist or physiotherapist with experience in post-viral fatigue syndrome. The cognitive function may be interrupted, and a further referral may be indicated for a more detailed assessment.

## 8. Conclusions

Patients recovering from COVID-19 may present with significant parenchymal, functional, and physiological abnormalities persisting for several months following primary infection. Radiographic patterns may include non-fibrotic and fibrotic-like features, while diffusion capacity reduction and a restrictive pattern are the most common respiratory function abnormalities. Although the nature of these post-acute consequences has been well described, much less is known regarding optimal management strategies including pharmacologic and non-pharmacologic interventions. Currently, ongoing clinical trials may provide evidence on the therapeutic potential of short-term corticosteroid and anti-fibrotic regimens in highly characterized subgroups of patients. Various national and international medical associations suggest that patients who have recovered from COVID-19 and present with limitation of their physical activity, reduction of their quality of life, and/or associated symptoms including shortness of breath, fatigue, pain, and/or weakness of the upper and lower extremities may benefit from inclusion in pulmonary rehabilitation programs. The optimal structure and duration of these programs and the most appropriate time point of implementation, remain to be determined in the context of future, large, randomized controlled studies. An evidence-based multi-disciplinary team approach for phenotypic characterization of those individuals who will most likely benefit from timely therapeutic interventions is sorely needed.
